# Cranial ultrasonography in diagnosis of infantile cerebral cavernous malformation

**DOI:** 10.1002/ccr3.7152

**Published:** 2023-03-27

**Authors:** Shuya Kaneko, Iku Ikeno, Hideo Wada

**Affiliations:** ^1^ Department of Pediatrics Noto General Hospital Nanao Ishikawa Japan; ^2^ Present address: Department of Pediatrics and Developmental Biology, Graduate School of Medical and Dental Sciences Tokyo Medical and Dental University Tokyo Japan

**Keywords:** cerebral cavernous malformation, cranial ultrasonography

## Abstract

The case of an infantile cerebral cavernous malformation detected by transfontanelle cranial ultrasonography. Infantile cerebral cavernous malformations tend to cause more major bleeding compared to those in older groups, so early detection and treatment are crucial. Cranial ultrasonography can contribute to the early diagnosis of infantile cerebral cavernous malformations.

## Graphical abstract

The lesion of cerebral cavernous malfoemations detected by transfontanelle ultrasonography.

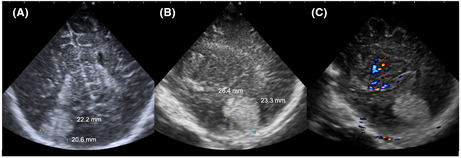

## 1 CASE PRESENTATION

**FIGURE 1:**
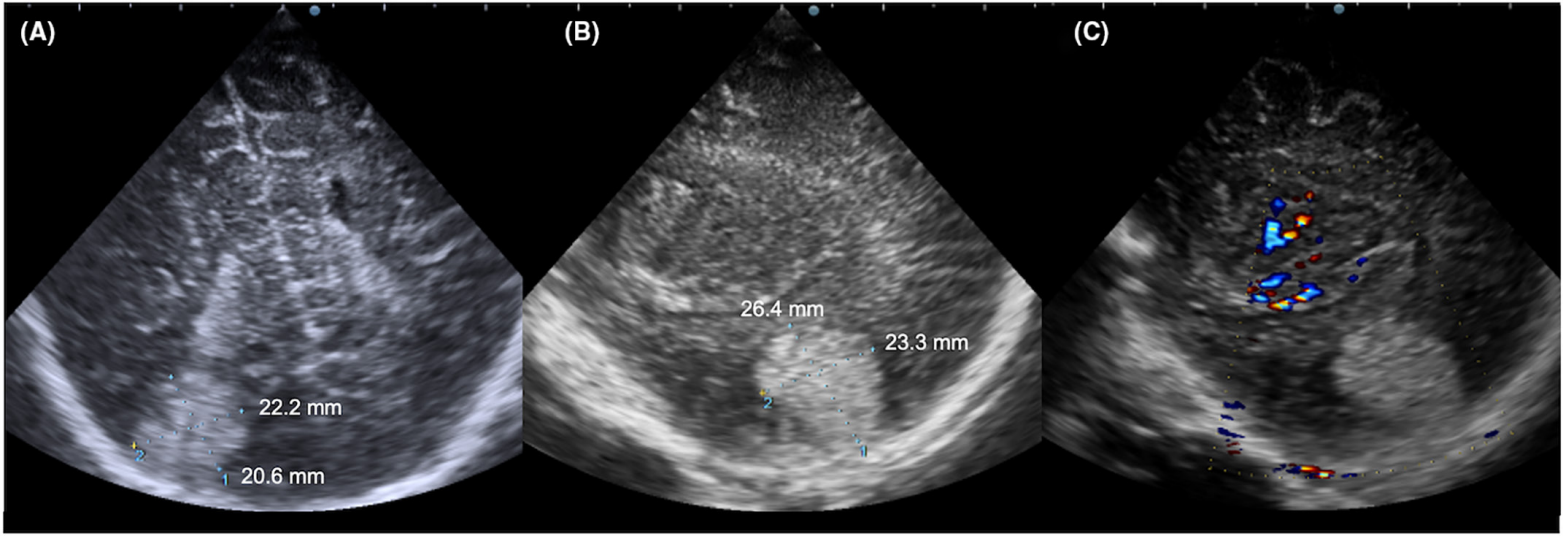
Transfontanelle ultrasonography. (A) Axial view. (B) Sagittal view. (C) Sagittal view with color Doppler. The lesion was located in the right occipitotemporal region. No flow signal was detected in the lesion. The lesion measured 20.6 × 22.2 mm in the axial view and 26.4 × 23.3 mm in the sagittal view.

**FIGURE 2:**
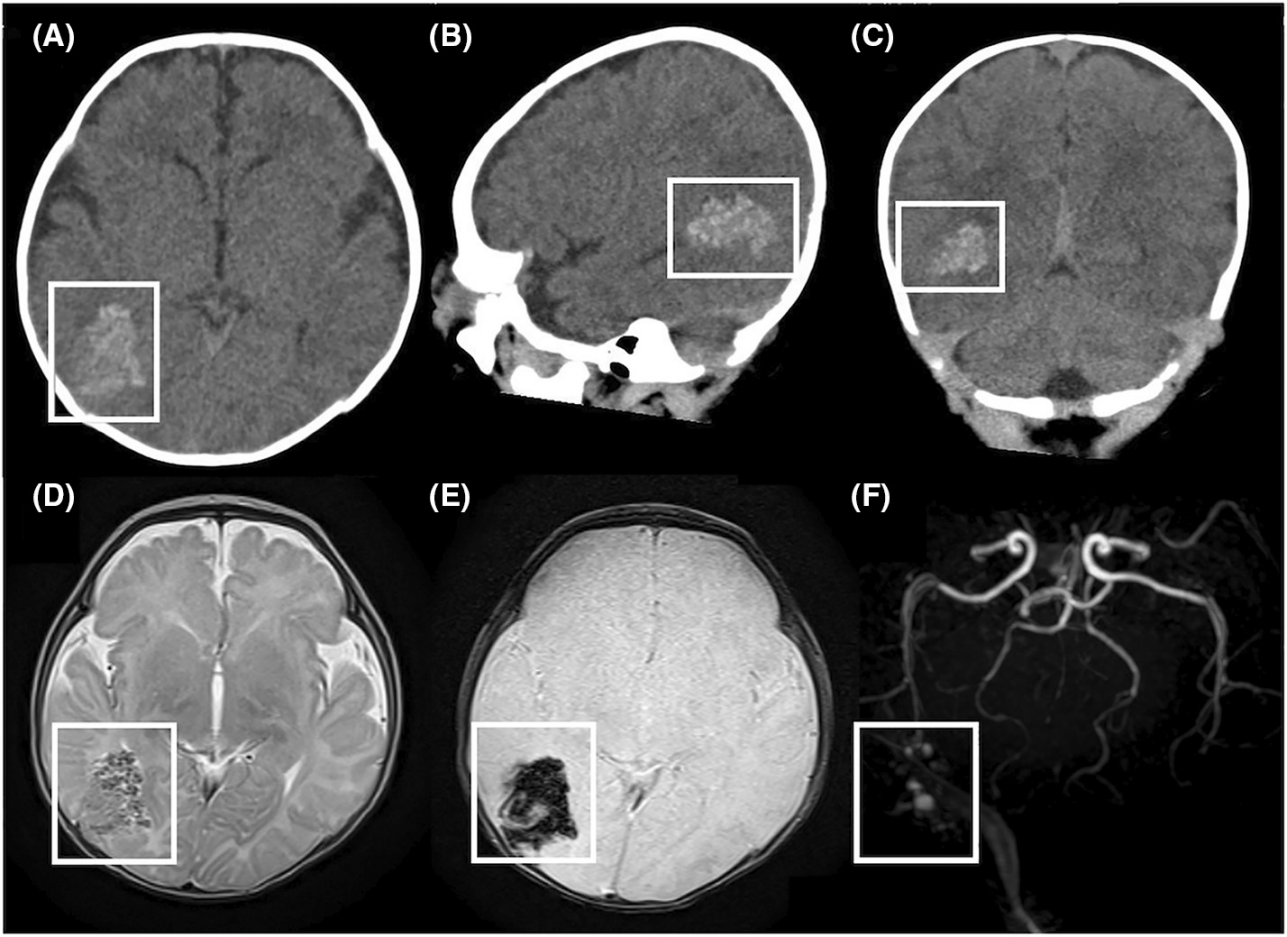
(A–C) Computed tomography. (A) Axial view. (B) Sagittal view. (C) Coronal view. The lesion was hyperdense and located in the right occipitotemporal region (white box). (D–F) Magnetic resonance imaging. (D) T2‐weighted sequence. (E) T2‐star‐weighted sequence. (F) Magnetic resonance angiography. The lesion appeared as a cluster of low signal‐intensity nodules on T2‐weighted images and a low signal‐intensity lesion on T2‐star‐weighted images. No obvious feeder vessel was identified on magnetic resonance angiography.

A 1‐month‐old boy presented with a 2‐day history of cough, runny nose, and fever. A rapid respiratory syncytial virus antigen test was positive and he was admitted to the hospital for treatment. Although chest auscultation revealed a systolic heart murmur, cardiac ultrasonography showed no abnormality. Ultrasonography of the cranium revealed a hyperechogenic lesion in the right occipitotemporal region suspicious for hemorrhage (Figure 1). Computed tomography of the head showed a hyperdense lesion in the same area (Figure 2A–C) that appeared as a cluster of low signal‐intensity nodules on T2‐weighted magnetic resonance imaging (Figure 2D–F). Therefore, arteriovenous malformation or cavernous malformation was suspected and he was transferred to a tertiary care hospital. Eventually, cavernous malformation was diagnosed. Surgical resection was later performed because of lesion enlargement and bleeding during follow‐up. In the 2 years since surgery, he has shown normal growth and development without neurological sequelae.

## 2 DISCUSSION

Cerebral cavernous malformations are enlarged vascular channels in the brain parenchyma lined by a single layer of endothelial cells. Most are diagnosed with magnetic resonance imaging.^1^ Occurrence in children is uncommon, with reported prevalence rates ranging from 0.30% to 0.53%. However, 25% of children with a cerebral cavernous malformation will experience epilepsy, hemiplegia, headache, other symptoms, or multiple episodes of acute bleeding, which is associated with high rates of mortality and disability.^2^ Infantile cerebral cavernous malformations frequently cause major bleeding because the infant brain is vulnerable, which leads to higher morbidity compared with older patients.^3^ Therefore, early detection and diagnosis are crucial. Transfontanelle ultrasonography can be performed at the bedside without sedation even in infants, unlike computed tomography and magnetic resonance imaging. Cranial ultrasonography can contribute to early diagnosis and a good prognosis for patients with infantile cerebral cavernous malformations.

## AUTHOR CONTRIBUTIONS

**Shuya Kaneko:** Conceptualization; data curation; investigation; writing – original draft. **Iku Ikeno:** Supervision. **Hideo Wada:** Supervision; writing – review and editing.

## FUNDING INFORMATION

This research did not receive any specific grant from funding agencies in the public, commercial, or not‐for‐profit sectors.

## CONFLICT OF INTEREST STATEMENT

The authors have no conflicts of interest to declare.

## CONSENT

The patient's parents provided written consent for publication of this case report and the accompanying images.

## Data Availability

The data that support the findings of this study are available from the corresponding author upon reasonable request.
